# Improving quality of care through routine, successful implementation of evidence-based practice at the bedside: an organizational case study protocol using the Pettigrew and Whipp model of strategic change

**DOI:** 10.1186/1748-5908-2-3

**Published:** 2007-01-31

**Authors:** Cheryl B Stetler, Judith Ritchie, Joanne Rycroft-Malone, Alyce Schultz, Martin Charns

**Affiliations:** 1 Health Services Department, Boston University School of Public Health, Boston, MA, USA; (office) 321 Middle St., Amherst, MA 01002, USA; 2 McGill University Health Centre, Montreal, Quebec, CA; 3 Centre for Health-Related Research, University of Wales, Bangor, UK; 4 Center for Advancement of Evidence-based Practice, Arizona State University, Tempe, AZ, USA; 5 Veterans Administration HSR&D Center for Organization, Leadership & Management Research, Boston, MA, USA; Program on Health Policy & Management, Health Services Department, Boston University School of Public Health, Boston, MA, USA

## Abstract

**Background:**

Evidence-based practice (EBP) is an expected approach to improving the quality of patient care and service delivery in health care systems internationally that is yet to be realized. Given the current evidence-practice gap, numerous authors describe barriers to achieving EBP. One recurrently identified barrier is the setting or context of practice, which is likewise cited as a potential part of the solution to the gap. The purpose of this study is to identify key contextual elements and related strategic processes in organizations that find and use evidence at multiple levels, in an ongoing, integrated fashion, in contrast to those that do not.

**Methods:**

The core theoretical framework for this multi-method explanatory case study is Pettigrew and Whipp's Content, Context, and Process model of strategic change. This framework focuses data collection on three entities: the Why of strategic change, the What of strategic change, and the How of strategic change, in this case related to implementation and normalization of EBP. The data collection plan, designed to capture relevant organizational context and related outcomes, focuses on eight interrelated factors said to characterize a *receptive *context. Selective, purposive sampling will provide contrasting results between two cases (departments of nursing) and three embedded units in each. Data collection methods will include quantitative tools (e.g., regarding culture) and qualitative approaches including focus groups, interviews, and documents review (e.g., regarding integration and “success”) relevant to the EBP initiative.

**Discussion:**

This study should provide information regarding contextual elements and related strategic processes key to successful implementation and sustainability of EBP, specifically in terms of a pervasive pattern in an acute care hospital-based health care setting. Additionally, this study will identify key contextual elements that differentiate successful implementation and sustainability of EBP efforts, both within varying levels of a hospital-based clinical setting and across similar hospital settings interested in EBP.

## Background

Evidence-based practice (EBP) is currently an expected approach to improving the quality of patient care and service delivery in health care systems internationally. It has been a widespread expectation for a number of years, but is yet to be realized. Numerous authors note the gap between current practice and available evidence and/or describe multiple barriers to achieving EBP [1-8]. One barrier that is recurrently identified is the setting or context of practice. Context is likewise cited by some as a potential part of the solution to the evidence-practice gap [6,9-12].

The Committee on Quality of Health Care in America, in *Crossing the Quality Chasm*, noted the need to recognize *quality *as a *system *property, that is, as a *contextual *property. This need includes systems that "facilitate the application of scientific knowledge to practice, and provide clinicians with the tools and supports necessary to deliver evidence-based care consistently and safely [p. 7–8, [1]]." Such a focus inherently implies the necessity of a broad, strategic view of the practice environment relative to EBP, or, as evolving research suggests, a need to consider methods and strategies for integrating use of evidence into the fabric of the clinical organization [9,10,13,14]. Such an EBP *normalization *or *institutionalization *approach is not evident in most organizations, nor is it the primary focus of implementation research. Instead, there appears to be a narrow project-, practice-, standard-, guideline-, or procedure-oriented approach to introducing evidence for the purpose of improving the way care is delivered in clinical settings. The same narrow approach appears to exist for studying the related implementation process. This fragmented focus has not sufficiently enhanced our knowledge of sustainable implementation. Neither has it appeared to consistently spread related improvements, if they are initially achieved, and thus the research-practice gap continues to be a challenge.

Evolving science in the area of EBP supports the critical role of context, that is the critical role of the health care environment in which practice and EBP efforts take place. Despite this evolving knowledge, it is unclear exactly what key contextual elements are involved, how executives and other organizational leaders can achieve this contextual quality, and what organizational interventions might be tested by researchers to provide guidance to organizational leadership. This project will study the role and evolution of context in the routine or ongoing translation of evidence into practice within targeted services. The "targeted service" in this study will be departments of nursing – a critical player in quality in any health care organization.

### Research objectives and overview

The *purpose *of this project is to understand both key contextual elements and related strategic processes in organizations that find and use evidence at multiple levels – in an ongoing, integrated fashion – in contrast to those that do not. More specifically, it seeks to:

∘ Identify key contextual elements and related strategic processes relevant to successful implementation and sustainability of EBP as the *norm *within an acute care hospital setting; and

∘ Identify key contextual elements that differentiate successful implementation and sustainability of EBP efforts, both within varying levels of a hospital-based clinical setting and across similar hospital settings interested in EBP.

Table 1 provides definitions both underlying this purpose and relevant to other study components.

**Table 1:** Underlying study definitions

• **Context/organizational context**:
∘ Overall: The health care environment in which practice takes place and characterized by *organizational culture, leadership, basic organizational components, and type of clinical setting*.
∘ Pettigrew/Whipp[17]: An essential dimension or the WHY/motivation behind a strategic change to EBP.
• **Content**: One of Pettigrew/Whipp's essential dimensions, in this case the WHAT of strategic change; i.e., organizational elements or processes in the system changed to enhance or support the use of evidence.
• **Evidence based practice (EBP)**: Practice derived from the best available evidence to achieve positive outcomes. This practice may range on a continuum from implementing a discrete practice (e.g. consistently using an evidence-based scale to assess the situation and implementing research-based interventions) to consistent ways or patterns of decision-making and practice (e.g. consistently seeking the best evidence in all decision-making to achieve positive outcomes).
• **Evidence**: Knowledge derived from a variety of sources that has been subject to testing and has been found to be credible [67,68]. This includes:
∘ Research,
∘ Patient experiences and preferences, and
∘ Practical knowledge and local data (e.g. audit, quality assessments, planning and project data)
• **HOW of strategic change**: See Process.
• **Implementation**: Efforts designed to get evidence-based findings and related products into use via effective change interventions.
• **Infrastructure**: Organizational structures, systems, roles, processes, relations, alignments, and capabilities.
• **Institutionalization**: Integration of evidence-based practice into the routine fabric of the organization [10]; also known as normalization.
• **Intervention**: Method or technique to enhance change.
• **Levels within the institution/institutional levels**: Individual, group/team, organization, larger external system [38]. In this study, these levels refer to individual clinicians and leaders; EBP-related project teams or committees; clinical units; clusters of units within a service; department of nursing; hospital; and external health care-related environment.
• **Norm or Routine per EBP**: Integrated into the everyday work of the clinical setting, in the policies, in the practices, in documentation, in the infrastructure, etc.
• **Normalization**: It is the routine occurrence of EBP; see Institutionalization.
• **Process**: One of Pettigrew/Whipp's essential dimensions [17], in this case the HOW of strategic change; i.e., the methods, strategies, or implementation interventions used to try to enable the use of evidence.
• **Research utilization (RU)**: The systematic process of transferring research knowledge into practice for the purpose of understanding, validating, enhancing or changing practice. RU consists of both the use of products of research and use of the research process [69].
• **Receptive context for change**: "A combination of factors from both the inner and outer context that together determine an organization's ability to respond effectively and purposively to change. ... [p. 373, [11]." Per Pettigrew et al. [12].
• **Routine**: See Norm or Institutionalization.
• **"Strategic"**: Refers to planned, organizational approaches to change and its deliberate management.
• **Sustainability**: Changes (practice and outcomes) based on evidence that continue over time as related to specific projects.
• **WHAT of strategic change**: See Content.
• **WHY of strategic change**: See Context.

The current state of knowledge in this field suggests that it is premature to propose hypotheses or to use a research design to test hypotheses. Given the need to better understand specific organizational factors that are key to normalizing EBP, and the inherent complexity of such phenomena, this study will use an explanatory case study approach [15]. Case study research, built on study questions, will provide a rich description of relevant organizational phenomena. Following this descriptive and theoretical work, propositions can be developed for future testing.

The primary research questions for this case study are as follows:

1. What *key contextual elements *support and facilitate: a) Implementation of EBP at the project level, and b) Normalization of EBP within a health care system at multiple institutional levels?

2. What *implementation interventions *or *strategic processes *are used to: a) Facilitate implementation at the project level, and b) Create normalization of EBP within a health care system at multiple institutional levels?

In this study, the term *context *is defined as the local health care environment in which practice takes place, including related organizational elements (see Table 1) [16]. Additionally, within our conceptual framework the term context is one of Pettigrew and Whipp's three "essential dimensions" of strategic change (i.e., "content, context and process") [17]. Related definitions are explained more fully below.

Our theoretically-based data collection will also afford a post hoc opportunity for exploration of three EBP models relevant to nursing as well as health care in general: the Ottawa Model of Research Use [18], PARIHS framework [9], and Stetler organizational model [10]. Each of these models is designed to provide guidance on how to achieve successful implementation. Each has a contextual element, and study data will be used to scrutinize those contextual elements.

### Significance and rationale

Implementation of available evidence into practice is a critical issue, as a great deal of research has little or no impact on practice [2-5]. Simply 'pushing out' evidence to caregivers through written documents or education has only limited success; and rather than a simple linear and logical process, studies are demonstrating that implementation efforts are messy and challenging [11,19]. Furthermore, relevant research has recognized that the process of implementation most often takes place within an organizational context that can have either a facilitative or hindering impact upon the adoption of research findings [10,11,16].

Another critical implementation issue is the frequent and often negative or narrow focus of research regarding organizational factors, such as the focus on *barriers *to use of *individual *targeted evidence in *time-limited projects*. Little research has been conducted on what contextual factors might be essential to *enable *the repeated, ongoing, *routine *uptake of evidence, or on the strategic management processes that could *facilitate *a change to support "*normalized*" EBP [10,11]. Within the hospital setting, the department/directorate of nursing offers a structured series of levels in which to study the concept of organizational/normalized EBP. Given its typical role in management of patient care units and related resource allocation, nursing is increasingly recognized as pivotal both to the quality of care in general and to the implementation of interdisciplinary-based quality care [20]. Importantly, the nursing profession also has a long history with "research utilization" (RU) [21-23].

In the service setting, RU, and now the broader but related concept of EBP, has long been stated as a goal for nursing departments. Over the years a number of nursing departments have described such efforts at implementation [24-27]. Several have achieved that goal to such an extent that they have become recognized internationally [28,29]. However, it is unlikely that the self-reported information available in the literature about such "best practice" departments adequately explicates the complex contextual factors and strategic processes needed to replicate successful implementation. Therefore, this study focuses on examining how organizations "make it happen." More specifically, it focuses on the explicit and replicable HOW, WHY and WHAT of the context that helps an organization to successfully implement and sustain EBP as a pervasive pattern (see Table 1). Because little, if any research has been conducted to understand the relationship among organizational context, related strategic management decisions, and the reported success of such EBP efforts in nursing, this study will contribute to our transferable and pragmatic understanding of such an important issue.

### Overview of literature

Early lessons about translating findings into practice are being called into question based on more recent reviews and evolving research [19,30,31]. There are now calls both for better theoretical underpinnings for implementation interventions at the individual provider level and for better information about the critical influence of organizational context [11,19,32]. Relevant to this study, Greenhalgh et al.'s extensive review of literature on diffusion of innovations in service organizations specifically calls for more research on "how organisations might create and sustain an absorptive capacity for new knowledge and ...achieve ... key components of a receptive context for change [11]." Fixsen et al., in a more recent review of implementation research across multiple disciplines, further notes the importance of organizational context and the fact that "facilitative administration is often discussed and rarely evaluated with respect to implementation outcomes [6]."

In most implementation research in health care, where change efforts have primarily focused on physician-provider behavior, there is growing evidence that the organization plays a key role in implementation results. For example, Bradley et al. studied hospital efforts to improve use of β-blockers [30]. They found that the presence of shared goals for quality improvement (QI), use and availability of credible feedback data for monitoring improvements, and the degree of support from hospital administration and clinical leadership – per related advocacy for the EBP – were key factors in differentiating high versus low performance.

Scientific studies about the influence of organizational context on the *routine *implementation of EBP are limited in general and in nursing specifically [33]. Much of the prior research in nursing has consisted of surveys on the perception of barriers to RU. Related findings have consistently indicated that nurses often view characteristics of the organization, akin to our definition of context, as a barrier [34,35]. However, the *BARRIERS to Research Utilization Scale*, which is most frequently used in such surveys, provides only a limited view of *context *through its eight related items [36]. A more recent descriptive study involving nurses assessed the degree of perceived *organizational support *for RU. They found that more RU was reported on units that also reported more "people support, positive attitude towards research utilization among the management, and organizational support [37]."

Ferlie and Shortell [38], after assessing initiatives on the quality of health care in the United Kingdom and the United States – which assumedly includes EBP, suggested that organizations need to recognize the key role of context, specifically in terms of a set of "core" elements: 1) organizational culture that supports learning throughout the care process, 2) leadership at all levels, 3) emphasis on the development of effective teams, and 4) greater use of information technologies for continuous improvement and external accountability. The elements of culture, leadership, and teamwork/collaboration also have been identified in the EBP literature. For example, in a concept analysis by McCormack, et al. [16], as well as in individual studies and various reviews of the literature, the potential importance of *culture *on adoption behavior is cited [11,12,31,37,39]. A case in point is a set of case studies regarding use of evidence in four types of multi-system clinical programs, which found that "the speed of adoption is influenced by the degree to which the innovation requires changes in organizational culture [31]." In terms of leadership, Greenhalgh et al.[11] and Estabrooks et al., among others, found leadership to be important to adoption/RU/EBP [11,37]. Greenhalgh et al.'s synthesis, for example, suggested that leadership was one of five "broad determinants" of organizational innovativeness – again strongly linked to the determinant of a receptive culture [11]. Other studies have identified the potential importance of teamwork and collaboration [11,40].

Unfortunately, the precise aspects of culture that are important to EBP are yet to be substantiated, and there is no consistency in "leadership" definitions. Research on leadership has often focused more on the characteristics of a leader than the types of behaviors that make a difference in successful implementation, or more importantly for this study, in institutionalization [11]. The above studies suggest the importance of various factors, but without the level of detail needed for EBP-related organizational interventions.

Two other *contextual factors *of potential significance are organizational infrastructure and unit variability. Infrastructure is defined broadly as organizational structures, systems, roles, processes, relations, alignments and capabilities. A few examples of the specific aspects of infrastructure that have been suggested as important to implementation, either in the innovation literature or within specific EBP studies, include: effective monitoring and feedback systems and, as with Ferlie and Shortell's core elements [38], related information technologies [11,30]; external communication networks and boundary-spanning roles [11,41]; and a defined organizational approach to "change" projects, a project lead, a facilitator, and coordinative mechanisms across departments or disciplines [2,31,42-44]. In terms of general variability at the level of organizational units, it is unlikely that all units within a given service will reflect the same "context" or degree of specific contextual factors [45]. In terms of EBP specifically, there is some evidence to suggest that unit level factors such as access to computers, organizational slack, autonomy, leadership style, or the quality of relationships and interactions – such as the degree of harmony between leaders and staff, may influence nurses' use of research evidence [46-49]. These factors, along with other unit-relevant contextual influences, require further study.

In conclusion, when a general innovation or a new EBP is introduced into an organization, a change process is assumedly involved. If an organization is to make EBP the routine approach to practice, it appears unlikely to occur without strategic change and the related management of key contextual elements. An understanding of both organizational change and elements of context *specifically related to EBP *are thus critical to success in normalization. As Greenhalgh et al. indicate, however, "the evidence on implementation and sustainability ... [which is] difficult to disentangle from that on change management and organizational development in general," is an under-researched area [11]. As a result, little guidance exists for nurse executives or others in health care administration regarding either which specific contextual elements are important or the strategic change/management processes needed to move an organization toward EBP as the norm. Further, discussion regarding organizational factors in EBP studies, often done retrospectively, has frequently related to the use of individual targeted evidence in time-limited projects, regarding individual clinicians, and involving isolated policies/procedures. Additional research needs to focus on contextual factors within a broader frame of reference relevant to the *routine *uptake of evidence across various organizational levels (Table 1). Such research also is needed to better understand how facilitative or receptive contexts emerge or are developed, in order to better inform and guide executives interested in this critical area of health care.

### Theoretical framework

Given the current state of science, a key assumption underlying this case study is that organizational change is integral to the achievement of, ongoing success with, and sustainability of *routine *EBP [38,50]. Where such routine EBP does exist, it is assumed that at some point in time certain "receptive" conditions were created – that is, change took place to enable EBP to become the norm [11,50,51]. It may be that some of these conditions were put in place in the past for other reasons, while additional conditions had to be introduced more recently and deliberately for EBP. It is further assumed, based on research literature on organizational change, that such change has to be led and strategically managed [52-54]. A final assumption is that such change is highly complex, and its study must account for significant dynamics within the change process relative to multiple levels within an institution [11,13,17].

The theoretical framework for this explanatory case approach is Pettigrew and Whipp's Content, Context, and Process model of strategic change [17], or more specifically the *strategic management of change *[13]. This model has been "widely used in analyzing and learning retrospectively from change programmes in organizations" and was based on empirical case-based organizational research [p. 33 [41]]. Although originally developed to understand competitive private sector organizations, it was later applied to a study of health care [12].

Users of the Pettigrew and Whipp model's three "essential dimensions" of strategic change (i.e., "content, context, and process") may interpret each term in slightly different ways [17]. However, overall the model focuses researchers and managers on the *WHY *of strategic change with relevance to *context*; the *WHAT *of strategic change in terms of its *content*; and the *HOW *of strategic change *processes*. When applied to health care by Pettigrew et al., the overall framework helped to identify several factors related to more successful strategic change [12]. These factors or "signs and symptoms of receptivity" include the following: quality and coherence of policy; key people leading change; supportive organizational culture, including the managerial subculture; environmental pressure; good managerial and clinical relations; co-operative inter-organizational networks; a fit between the change agenda and its locale; and the simplicity and clarity of organizational goals and priorities [12,44]. These factors are dynamically linked and form a pattern receptive to the desired change or innovation. However, there is no apparent common, exact path or recipe by which these common factors come together to create success [17,51].

Given the differential views available regarding the meaning of each of the three overarching dimensions of the framework (*context*, *content *and *process*) and their related operational counterparts (the *why*, *what *and *how *of change, respectively), it became imperative to clearly articulate definitions underlying the study. This was important in terms of both relating the individual dimensions to the signs and symptoms (S&S) of receptivity, and identifying and creating detailed data collection tools. Table 1 articulates our definition of each of the framework's dimensions. Table 2 illustrates how those dimensions in turn are perceived to relate to individual S&S of an overall *receptive context *– in terms of our broader meaning of context – and to our overall approach to data collection. Table 2 reflects the fact that S&S may emerge at different times (playing different functions/multiple dimensions) over the dynamic life of an organization and its related change. This reinforces the fluid nature of the dimensions, the signs, and their inter-relationship – the pattern of which may vary from organization to organization and within organizations.

**Table 2:** Relationships between Pettigrew et al. framework and data collection approaches [13, 17, 51]

"Pettigrew" Essential Dimensions/Questions	Signs and Symptoms/Characteristics of Receptive Contexts	Data Collection Approaches/Tools (Across Characteristics)	Level of participants	Specific Question Examples (Will always explore both targeted or single EBP change and broad EBP change across a case's timeline)**
**WHY (Context, relative to motivation for strategic change toward EBP):** • *Why do nursing departments/directorates, and their embedded levels, wish to/implement EBP?*	• Environmental pressure • Supportive organizational culture • Key people leading change	1. Individual Interviews & Focus groups: a. Motivation b. Driving or restraining forces 2. Surveys a. Goh's Org. [58] Learning Survey b. MLQ Leadership Tool [59] c. NWI [60] 3. Document Review	1. Unit leaders 2. Unit staff 3. Hospital leadership 4. Relevant project or committee staff	1. What was the motivation for change: ∘ Why did unit/hospital wish to implement EBP (specific project; general approach)? 2. What enabling/driving or restraining/hindering forces over time influenced that motivation (internal and external environment)?

**WHAT (Content, relative to organizational elements or processes in the system changed to enhance or support the use of evidence):** • *What changes are made relative to key contextual elements to enable implementation and/or routine EBP?*	• Quality and coherence of policy, e.g., alignment/infrastructure • Managerial-clinical relations (e.g., team building) • Supportive organizational culture • Cooperative inter-org networks • Key people leading change	1. Individual Interviews & Focus Groups 2. Surveys a. NWI [60] b. Goh's Org. Learning Survey [58] 3. Document review	1. Unit leaders 2. Unit staff 3. Hospital leadership 4. Relevant project or committee staff	1. What was the content of the change at the project level, e.g., what in the system was changed to enhance, support and sustain use of an individual, targeted piece of evidence? 2. What was the content of related contextual change for generic, sustained EBP over time, e.g., what key organizational structures, systems, roles, etc. were changed to enhance or support routine use of evidence?

**HOW (Process, relative to methods, strategies, or implementation interventions used to try to enable the use of evidence)**: • *How do nursing departments/directorates, and their embedded levels, get EBP implemented ... including on a routine basis?* • *How and which implementation and other change strategies are used to achieve change at both the individual team and organizational levels relative to successful and sustained implementation of EBP?*	• Quality and coherence of policy (e.g., use of evidence) • Key people leading change (e.g., with appropriate skills) • Cooperative inter-org networks • Simplicity and clarity of goals • Change agenda & its locale	1. Individual Interviews & Focus Groups 2. Document review 3. Targeted group observations	1. Unit leaders 2. Unit staff 3. Hospital leadership 4. Relevant project or committee staff	1. What processes were used to enhance an individual targeted change to EBP, e.g., what implementation interventions were used to encourage adoption of the change? 2. What strategies were used over time to facilitate a change to EBP as the norm? Examples might include nurse manager EBP rounds, targeted leadership retreats, use of an external consultant in EBP, and special communication methods/media focused on EBP and its value.

**Some of the receptive characteristics may be pre-existent when an innovation or vision is proposed, having evolved overtime; or, new conditions may need to be created for innovation to succeed. Thus characteristics may in fact be found under more than one of the major study questions of what, why and how.

Once the above conceptual perspectives were articulated, more detailed definitions of the S&S were needed in order to direct specific data collection efforts. As with the essential dimensions, the essence of various signs and symptoms was not always transparent in light of the study's focus on institutionalization of EBP. Therefore, building on existing descriptions of organizationally-related elements relevant to each receptivity factor [11,12,44], the following supplemental sources were used to facilitate development of each factor's operational definition:

▪ EBP models that include a contextual element or focus [9,10,18], and

▪ Literature on implementation interventions and organizational innovation, particularly as reflected in our **Overview **discussion.[6,7,11,19,30-32,39,43,55,56].

These supplemental resources were useful in clarifying operational definitions of the potential HOWs and WHATs of strategic change and its management, particularly for the *Change Agenda *and *Quality & Coherence *factors. See Table 4, as well as the additional files, which illustrate use of these supplemental sources. [See Additional file 1] [See Additional file 2]

**Table 3:** Core analytical general and specific research questions: Key contextual elements

What *key contextual elements *support and facilitate a) implementation of EBP at the project level and b) normalization of EBP within a health care system at multiple institutional levels?
1. Do key contextual elements differentiate successful implementation, as well as sustainability of EBP efforts, from less successful efforts within varying levels of a hospital-based health care setting?
• In terms of elements either pre-existent or created through strategic change.
• In light of the interrelationship of key contextual elements over time.
2. Do key contextual elements differentiate successful implementation and sustainability of evidence-based practice efforts from less successful efforts across similar health care settings interested in EBP?
3. Does the number of embedded units (i.e., a critical mass) within a service (and services within a department) with key contextual elements influence the extent to which an organization has successfully implemented and sustained evidence-based practice at both a project level and as the norm at multiple institutional levels?
4. To what extent does each of the identified models of RU/EBP reflect the key contextual elements identified in this study and the literature as relevant to successful and sustained implementation of EBP?

**Table 4:** Core operational research question and sample related sub-questions: Implementation interventions and strategic processes

What *strategic approaches *or *implementation interventions *are used to a) facilitate implementation at the project level and b) create normalization of EBP within a health care system at multiple institutional levels?
1. **WHY: **What was/were the specific *motivation/s *for change/s, i.e., why did targeted departments/services and their embedded levels wish to/implement EBP?
i. In terms of specific projects.
ii. In general, within the department/service and other embedded levels.
2. **HOW: **What was the *process *used to create an individual change to EBP, i.e., what was the method used to try to get EBP implemented?
i. Which, if any, specific implementation interventions/strategies were used to try to enable the use of an individual, targeted piece or program of evidence?
▪ *E.g., use of a dedicated project lead? Use of a standard organizational approach to change project? Use of a facilitator/champion? Use of E-B change strategies, e.g., audit/feedback, opinion leadership, QI team, clinical reminder, etc.?*
3. **WHAT: **What was the *content *of related contextual change for generic, sustained EBP over time?
i. What key contextual elements or other entities in the system were changed to enhance or support the routine use of evidence?
▪ E.g., alignment of infrastructure with the new purpose, values, vision, strategy, priorities ...i.e., change in various operational structures, systems, roles, job descriptions, processes, and relations; budgeting; etc.

## Methods

This is a multi-method explanatory case study. A case study approach is the method of choice, given our descriptive purpose, research questions, the complexity of organizational phenomena, and current state of knowledge in this field [15]. Our conceptual framework focuses data elements and collection approaches on a series of sub-questions. Our sampling method is designed to provide: a) an exemplar of the WHY, WHAT and HOW in a case known to have normalized EBP to a greater degree than others, and b) for contrast, a case just beginning the journey to institutionalization. Within each case, embedded levels will provide additional, comparative data. Each of these study elements is described below, along with other procedural details and our approach to analysis.

### Operational study questions

Sub-questions are built on our two primary study questions, the three entities of the *Why/What/How *of strategic change, and our conceptual sources regarding S&S of receptivity. The first primary question is a macro, analytical question (Table 3) focusing on theoretical explanation building and is being addressed through triangulation of all study data, e.g., from surveys and interviews. It is broken down into conceptual sub-questions (Table 3).

The second primary question is the operational question (Table 4), also broken down into sub-questions. The full set of operational questions is provided in a supplemental file. [See Additional file 1]. This document includes the foci of questions for individual interviews, focus groups, and group observation meetings. Actual interview questions will be based on this document and adapted to the targeted group and interview time. The bulleted examples, within the final level of sub-questions in Table 4 and the supplemental file [See Additional file 1], are for clarifying purposes and serve as the source of items for a set of stimulated recall checklists noted below.

In some cases key contextual elements may already exist prior to efforts to initiate EBP. These may be uncovered through questions relative to enabling conditions, reference to organizational history, and, for the beginning case, our survey data. Questions regarding enabling and hindering forces are also used to capture unanticipated factors or elements. Finally, operational questions reflect the study's focus on multiple levels within the institution.

### Sample and Recruitment

The study is being conducted in the United States. A case is defined as a department of nursing within a hospital. Such departments have an ordered series of levels that can be studied, as described in Table 1. Within each case, three embedded units will be selected.

In order to illuminate the research aims and assist in explanation building, purposive case sampling will be used. One case will be selected after a nomination process involving the American Organization of Nurse Executives (AONE), whereby a list of institutions perceived as exhibiting a high, sustained, normalized level of EBP are identified. The potential set of beginning cases will be recruited from members of AONE who self-report being "early in the journey to institutionalization." Final selections will be made by the team per top ranking (role model), self-rating of institutionalization (with rationale), interest in EBP and the study, feasibility for data collection, and the degree of matching hospital characteristics.

This selective, purposive sampling approach will provide contrasting results for predicable reasons [15]. This will allow testing of a preliminary proposition developed from the literature review and conceptual framework by the study team: *Successful EBP nursing departments have key contextual elements in place and/or experience a strategic organizational change relative to key contextual/organizational elements to achieve EBP outcomes*.

### Embedded units sample

Three embedded units per case will be included given the criteria of feasibility, institutional burden, grant funding, and diversity of patient populations. As noted earlier, a degree of variability is expected to exist within any organization and thus within units across a department of nursing – although there may be less variability within a role model case [45]. However, because of feasibility issues, rather than attempt to search for a set of varied units across a spectrum of diversity within both sites, the decision was made to focus on instances of best achievement or positive beginning effort across units with different types and intensity of patient populations. The units will be selected, to the extent possible, at random from those identified by nursing leadership in each case site as being *highly evidence-based *or *interested in such activity*. We will attempt to sample a medical, surgical and ICU unit in each hospital, and stratify the available sample as needed. These embedded units should provide a reasonable cross section of clinical services within the institution.

### Individual level sample

Individuals invited to participate include the following: all staff on selected embedded units; "leadership" in the form of all managers within the nursing department as well as clinical resource and/or specialty nurses, members of the quality management structure (within and outside of nursing), institutional senior level managers responsible for EBP, and other site-identified individuals said to be key to EBP; and, finally, participating members of three or four group meetings relevant to EBP. Stratified random selection will be used according to relevant categories, if needed, per availability of large numbers. However, selected individual leadership participants (for interviews) and groups (for observations) will be purposively sampled (e.g., the CNO, EBP project groups and their leads, and the procedure committee). Both nursing and interdisciplinary groups will be recruited. In terms of the interviews, approximately 20 individual interviews per case will be conducted, while approximately 400 subjects in total are expected to participate in various data collection activities. The number will vary depending on availability of potential participants, size of the organization and degree to which the data obtained becomes repetitive, with little new information emerging.

### Data collection

Data collection methods will include quantitative tools and qualitative approaches.

#### Quantitative tools

Four instruments with acceptable levels of reliability and validity will be used. First is the Research Utilization Questionnaire, adapted from Estabrooks' original tool to assess the extent of direct, indirect, and persuasive use of research in practice [57] [Personal communication, C. Estabrooks, University of Alberta, 10/30/2006; current version unpublished.] The three other study tools assess the nature of organizational elements identified as potentially critical, both within the Pettigrew framework and current implementation science (see Table 2) [6,11,12,17,51]. This includes Goh and Richard's Organizational Learning Survey (OLS) [58], judged by the team as assessing culture in a focused manner relevant to EBP [38]; the Multi-dimensional Leader Questionnaire for leadership assessment [59]; and the Nursing Work Index [60,61], which provides valuable information on collaboration/teamwork. The latter information also provides a baseline comparison of cases relative to their work environment for nursing practice [62]. These survey data will be collected about individual embedded units from nursing staff and about the nursing department as a whole from members of the hospital-wide nursing leadership team in each hospital. Following the Dillman approach, participants will receive a survey, then a reminder, and then a second survey.

#### Document review

Selected materials will be assessed for information regarding the degree to which use of evidence is integrated into the routine fabric of the organization, primarily per the Pettigrew et al. essential dimensions and S&S of a receptive context [17]. Sample documents include mission, philosophy and practice models, EBP project information, job descriptions and performance evaluation/appraisal forms or processes, and strategic approaches focused on EBP, such as communication vehicles, education/orientation content, and the like.

Documents also will be reviewed for indicators of success and maintenance of specific efforts. Internal, locally developed evidence and EBP outcomes will be explored, including report cards, QI summaries and project reports. Document reviews will provide primarily nominal, ordinal and qualitative data. A general description of the institution and its activities, per a public annual report, will be reviewed for background.

#### Observations

We will observe the meeting of three to four groups identified by site leadership as relevant to the EBP initiative and naturally occurring at the time of the site visit. Potential groups will include the procedure/standards/guideline committees and special EBP project committees. Such observations will provide investigators with a "live" example of EBP activity, thus adding supplementary insights about the organization. Immediately after the meeting, the investigator will record field notes regarding relevant processes that emerged, which will provide additional background as other structured data are analyzed. During the last 15 minutes of the meeting, the group will be asked brief questions to clarify and/or expand on issues and available documentation. Meeting questions are included in a supplemental file. [See Additional file 1]

#### Interviews

This data collection method will not only provide information regarding stakeholders' perspectives but also information unavailable from other sources. Interviews will be recorded and transcribed. Two types of interviews will be held, i.e., individual and group:

∘ *Staff nurses, within a group interview *– We will hold two to three, 45–60 minute focus groups of three to eight nurses on each of the three embedded units per site; and

∘ *Individual interviews with leaders *– Key stakeholders, as identified in the sample section above, will be interviewed for 60–90 minutes.

Within each type of interview, open-ended questions will be guided by the operational sub-question list [See Additional file 1]. In addition, participants will be asked, through a process of stimulated recall, about specific institutional and operational components based on the Pettigrew et al. framework and supplemental research [10,12,17,51,55]. [See Additional file 2] Through this process, we may unearth targeted conceptual-based data not previously identified; however, stimulated recall will be used only after participants have had an opportunity to provide spontaneous thoughts about the evolution of EBP.

#### Outcomes

*Success *in achieving EBP at multiple levels will be operationally defined in diverse ways, including the following:

1. The degree of EBP activity (at all levels) over time.

• Number of active EBP projects and number of units and related services engaged and making progress.

• Percent of polices that are current and substantiated with evidence.

• Percent of relevant procedures, protocols, practice assessment tools, etc. (the "Ps") that are evidence-based.

• Evidence of adherence to the "Ps" per audit and self-report.

2. The degree to which there is evidence (direct and perceptual) that *individual *targeted EBP projects' goals/objectives and outcomes were met.

3. Evidence regarding, and tracking of change in key nursing-sensitive outcomes, i.e., fall rate or patient self-care behavior. Such outcome data will be recorded in terms of comparative not raw terms in degree of improvement and at/above available benchmarks.

4. The degree to which there is evidence that needed strategic departmental changes per EBP-related goals/objectives were met.

5. Evidence of the status of the organization in relation to EBP.

• Self-ratings of staff on the research utilization tool [57].

• Concrete patient examples of nursing behaviors from caregiver/manager self-report that show episodic and/or "routine" use of evidence.

• Self-rating on "where their organization is on EBP." For example, are they *just starting to think about it*, *beginning to develop plans*, *making some progress*, *making good progress*, or *making very good progress?*

• Concrete managerial/leadership examples of behaviors from participant reports that show episodic or "routine" use and/or expectations of use of evidence in practice, i.e., use of EBP rounds [63].

*Maintenance *or the degree to which success has been sustained will be operationally defined as follows:

1. The degree to which there is evidence that identified EBP projects' targeted changes and related outcomes have been maintained over time, i.e., one-year post-implementation.

2. The degree to which there is evidence (both direct and perceptual) that identified strategic changes have been maintained over time, i.e., one-year post-implementation.

Applicable outcomes that have been achieved over the past three years will be sought including, as noted, ongoing use of evidence and sustainability of documented changes. Our various data collection methods will provide multiple views of potential outcomes, including self-reported "use," the perceived degree of success achieved in specific endeavors and overall normalization, and data-based results for targeted projects and indicators. Cumulatively, these data will be used to draw conclusions about project success/outcomes.

#### Procedures

The PI will conduct onsite visits of approximately eight days, and another investigator will assist her during a two-day visit at each site. A local facilitator with human subjects' protection training and familiarity with the organization, but not in a management position, will assist the work of the investigators.

It is highly likely that members of the role-model hospital, particularly leadership, will know of their widely recognized status. Initially, it may not however be clear how members of the beginning hospital view themselves. A number of hospital members may belong to AONE, and thus may have read the full study abstract. In any case, to mitigate the potential issue of socially desirable answers, to the extent possible, targeted recruitment and consent documents will indicate only that the two cases were chosen because both are highly interested in EBP and in making it part of the norm of practice. Specific recruitment and consent documents will not focus on the difference between the hospitals or emphasize the actual status of an individual site.

### Analyses

Data from this multi-method study will be summarized and compared to answer the study's analytical and operational questions (Table 3 and 4). Triangulation of the multi-method/multi-source data will be an essential element of the analysis. Overall, the process will be both deductive and inductive: i.e., deductive in that key terms and themes relative to the Pettigrew et al. model will be used as coding categories; and inductive to the extent that the investigators will be open to and will add unanticipated contextual themes identified relative to the evolution of EBP normalization and implementation [17].

Quantitative data will be analyzed according to the questionnaires' manuals using parametric and non-parametric techniques as appropriate within and across cases. Qualitative data will be subject to thematic content analysis following the procedure outlined by Miles and Huberman [64]. All qualitative data will be managed through NVivo. The ultimate description of each case will be based on the patterns that emerge from the quantitative data in surveys, mixed data from document reviews, and the primarily qualitative data from interviews, focus groups, and group meeting interviews/observations. As such, a pattern-matching logic, based on explanation building, will be used as a data analysis framework [15]

To enhance the study's trustworthiness, i.e., its credibility, transferability, dependability and the confirmability of our qualitative data and related interpretations, approaches identified by Lincoln and Guba [65] as well as Rycroft-Malone [66] for naturalistic inquiry will be used. This will include peer-debriefing at the site among the team's site visitors; checks with stakeholders regarding selected aspects of interpretation after preliminary analyses; an inquiry 'audit' by one of the investigators (MC) of the primary data collectors' documentation of methods, data, and decisions made during the collection and analysis process; and a "reflexive, methodologically self-critical account of how the research was conducted [66]." Also, to enhance reliability of the analysis of interviews, the first three interviews from each site will be coded by two investigators, compared for consistency, and discrepancies resolved through discussion and/or additional coding rule changes. A similar process will be conducted for the analysis of complex documents.

Consistent with case study methodology, each case is regarded as a 'whole study' in which convergent evidence is sought and then analyzed within data sets, within sites, across units and between staff and leadership, and, ultimately, across the two cases relative to the pre-identified key contextual factors [12,17,51]. Yin calls this cross-case analysis [15]. In this way, differences and similarities will be highlighted and comparisons made in order to assist theoretical explanation-building regarding the influence of context on the use and sustainability of EBP. In this way a more complete picture about the influence of context will be developed.

### Ethical considerations

The study was submitted to the Institutional Review Board at the Boston University Medical Campus (Boston, Massachusetts, USA). It was approved as Expedited. The protocol was subsequently approved by all other co-investigator sites and will be approved at each case study site. Neither the identity of individual participants nor the two cases will be revealed.

### Limitations

There are two, interconnected and potentially limiting factors, i.e., our sample size and the study's external validity. Financial and practical constraints dictated that only two sites be included. These two sites will be matched and thus involve only one size of hospital. While more sites would improve the credibility and statistical generalizability of various findings, the strength in use of a case study lies in the opportunity to collect multiple types of data enabling development of a comprehensive, in-depth picture of the influences of context on the routine use of evidence in practice. Thus, while findings of the study will have limited generalizability, they will be theoretically transferable and will lead to a number of propositions or hypotheses that can be tested in future research [15].

## Discussion

Data from this comparative, explanatory case study will be analyzed across data sources, across collection methods, across individual units within a case, and across case hospitals. This cross-case analysis and triangulation of all data results will enable us to highlight differences and similarities in order to assist explanation-building regarding the concept of a receptive context and related strategic change – specifically, in relation to the use and normalization of EBP. Overall, findings will provide a rich description. Following such descriptive and theoretical work, propositions can be developed that could be explored in further studies regarding the institutionalization of EBP. This could include action research for nurse executives and their implementation research partners interested in institutionalizing EBP. Other researchers could use results to formulate and test hypotheses using various methodologies. Finally, the summarized findings will be used to assess each of the three RU/EBP models as to their degree of inclusiveness of key contextual elements identified by the case study. These results will then be communicated to leadership audiences, along with our preliminary findings regarding the What, Why and How of strategically integrating EBP into the day-to-day fabric of care.

## Competing interests

The author(s) declare that they have no competing interests.

## Authors' contributions

CBS, JR, JR-M, and AS collectively developed the study plan with the support of a Canadian Institutes of Health Research International Opportunity Development/Planning grant. JR led the application for those CIHR funds. CBS conceived of the study, and lead the application for current grant funding, with key support from MC; she has drafted the initial form and final revision of this manuscript. All other authors (JR, JR-M, AS, and MC) have read drafted components of the manuscript, provided input into initial and final refinements of the total manuscript, and agreed to the final manuscript.

## Supplementary Material

Additional File 1 *CORE OPERATIONAL RESEARCH QUESTIONS: IMPLEMENTATION INTERVENTIONS AND STRATEGIC PROCESSES*. A listing, primarily within a table, of detailed operational research questions. Click here for file

Additional File 2 *STIMULATED RECALL SHEETS*. A set of checklists, primarily as a set of tables, to stimulate recall of key elements related to topics within the interviews. Click here for file
