# Supplementary material for: Improving quality of care through routine, successful implementation of evidence-based practice at the bedside: an organizational case study protocol using the Pettigrew and Whipp model of strategic change
Source: Implement Sci. 2007 Jan 31;2:3. doi: 10.1186/1748-5908-2-3 (PMC1803000; doi:10.1186/1748-5908-2-3)
Supplement: Additional File 1 — CORE OPERATIONAL RESEARCH QUESTIONS: IMPLEMENTATION INTERVENTIONS AND STRATEGIC PROCESSES. A listing, primarily within a table, of detailed operational research questions. [file 1748-5908-2-3-S1.pdf]

ADDITIONAL FILE 1: *CORE OPERATIONAL RESEARCH QUESTIONS: IMPLEMENTATION INTERVENTIONS AND STRATEGIC PROCESSES*

FOR INTERVIEWS & FOCUS GROUPS

The following lists the questions that will be the focus of individual interviews & focus groups. Staff questions will focus primarily but not exclusively on EBP project changes on the unit and leadership's primarily but not exclusively on EBP as a norm. Skilled interviewers will make slight wording changes appropriate for leadership vs. staff.

1. What *interventions or strategic approaches* are used to a) facilitate implementation at the project level and b) create normalization of EBP within a health care system at multiple institutional levels?
    a. **WHY:** What was the *motivation* for change/s, i.e., why did targeted departments/services and their embedded levels wish to/implement EBP?
        i. In terms of specific projects.
        ii. In general, within the department/service and embedded levels.
    b. **WHY:** What were the *enabling/driving forces and the restraining/hindering forces or factors*, over time, to the motivation to implement EBP?
        i. *Internal environment*: What was the *receptive capacity* (i.e., *key contextual elements*) to implement and sustain EBP, over time (including at the time of the decision to initiate EBP)? E.g.,
            - Strong leadership (Transformational)
            - Clear strategic vision
            - Good managerial relations
            - Visionary staff in key positions
            - Climate conducive to experimentation and risk-taking (Learning org.)
            - Effective monitoring and feedback systems
        ii. *External environment (for nursing, includes the hospital as a whole)*; e.g., regulatory mandate; Magnet interest
    c. **HOW:** What was the *process* used to create an individual change to EBP, i.e., what was the method used to try to get EBP get implemented?
        i. Which, if any, specific implementation interventions/strategies were used to try to enable the use of an individual, <u>targeted</u> piece or program of evidence?
            - *E.g., use of a dedicated project lead? Use of a standard organizational approach to change project? Use of a facilitator/champion? Use of E-B change strategies, e.g. audit/feedback, opinion leadership, QI team; clinical reminder, project plan, etc.?*
            - Did the processes/strategies that were used change over time?
        ii. How effective were the implementation efforts?
            - How did you measure the process and results? (Including E-B outcomes)
            - Were you able to overcome barriers and if so, how… e.g., attitudes, knowledge, lack of cooperation?
        iii. What were the *enabling/driving factors and the restraining/hindering factors*?
            - *E.g. receptive capacities (key people leading change), the organizational culture, teamwork, coordination across departments, and participation/involvement of staff, resources, time?*
            - E.g., other capacities like the availability of experts, trained facilitators, and external/internal evidence?
            - E.g., well substantiated and convincing internal and/or external evidence or a good plan?

a. **WHAT:** What was the *content* of related contextual change for targeted EBP projects over time?
    i. What in the system was changed to enhance or support the use of an individual, targeted piece of evidence?
        - E.g., policies, procedures, standards, performance or audit expectations?
        - E.g., improved relationships or a new operational system or role?
    ii. What were the *enabling/driving factors* and the *restraining/hindering factors*
    iii. Was successful implementation sustained?
    iv. How was it sustained?

b. **HOW:** What was the *process* used over time to create a change to EBP as the norm or to create "routine" EBP?
    i. Which, if any, strategies were used to try to enable the routine use of evidence?
        - *E.g., was there strategic visioning/planning/evaluation; focus on new capabilities, new beliefs, new language, and a new sense of purpose; identification of simple, clear and shared goals; use of a model or conceptual framework; leadership expectations for change; mandated changes; "marketing" or broad education or kick-off conferences; use of champions and opinion leaders; external networking; changeover of resistant individuals; leverage of external expectations; new language; special communication methods/media; coordinative mechanisms across departments or disciplines role modelling, use of consultants; demonstration projects; revision of priorities and protection of the EBP goal; ensuring the alignment of infrastructures with the goal of EBP; provision of resources and a new structure for implementation; engaging staff for broad based action; focusing on new, clearly articulated and supported values; generating short –term wins; consolidating gains and producing more change; celebrating wins and progress; special initiatives?*
    ii. How effective were these strategic implementation efforts, over time?
        - How was this assessed?
    iii. What were the *enabling/driving factors and the restraining/hindering factors?*

c. **WHAT:** What was the *content* of related contextual change for generic, sustained EBP over time:
    i. What key contextual elements or other entities in the system were changed to enhance or support the routine use of evidence?
        - *E.g., alignment of infrastructure with the new purpose, values, vision, strategy, priorities …i.e., change in various operational structures, systems, roles, job descriptions processes, and relations; budget; change in decision making structures and coordinative/collaborative mechanisms; formalization of new practices into policies, procedures and routine systems; change in internal communication and dissemination systems or external communication linkages; alignment of documentation; integration of new values/expectations into incentive systems; creation of monitoring/feedback systems & related information technologies, and focus of human resource training.*
    ii. What were the *enabling/driving factors* and the *restraining/hindering factors?*
    iii. Were related goals/objectives/outcomes met?
        - What is/has been the degree of EBP activity (at all levels) over time, its related success (per number of E-B outcomes); and its related maintenance (sustainability of activity & outcomes)?
    iv. Was successful transformational change sustained?
        - How was it sustained?

For Group Meeting Observation Questions

(Depending upon time and documentation available)

1. *Can you tell me about the progress you believe you are making in relation to your goals?*
2. *Given the degree of progress you are making, what do you think are the most critical factors that have:*
   a. *Facilitated your progress?*
   b. *Hindered your ability to move faster or further?*
3. *Exactly what did you do to try to get others to change?*
4. *What do you think the biggest challenge is for departments trying to create EBP as the norm?*
