# Supplementary material for: Improving quality of care through routine, successful implementation of evidence-based practice at the bedside: an organizational case study protocol using the Pettigrew and Whipp model of strategic change
Source: Implement Sci. 2007 Jan 31;2:3. doi: 10.1186/1748-5908-2-3 (PMC1803000; doi:10.1186/1748-5908-2-3)
Supplement: Additional File 2 — STIMULATED RECALL SHEETS. A set of checklists, primarily as a set of tables, to stimulate recall of key elements related to topics within the interviews. [file 1748-5908-2-3-S2.pdf]

ADDITIONAL FILE 2: *STIMULATED RECALL SHEETS*

1. PROJECT-RELATED CHANGE/HOW & WHAT: Intro by facilitator: *You have talked about the process you or others used to create an individual change to EBP in the ______ project. To jog your memory and make certain that we understand all of the implementation strategies used to try to enable the success of this project, could you please review this checklist and check off every approach you understand was used in this project (THERE IS NO RIGHT ANSWER). NOTE: The interviewer checks off and notes to the interviewee/s those heard during the prior part of the interview and then has the interviewee validate those. (May require interviewer to clarify meanings.)*

STRATEGIES USED TO ENHANCE THE ADOPTION OF AN IDENTIFIED, EVIDENCE-BASED PRACTICE per a formal PROJECT	*Please check "Yes" for all that apply to this particular project and then indicate whether you think they were useful or not*
Use of a dedicated project leader?	_____ *Yes:* _____*useful* ____*not useful*
Use of a standard organizational approach to change for the project?	_____ *Yes:* _____*useful* ____*not useful*
Use of a project facilitator/helper/guider? (If yes, was the person from the QI department?)	_____ *Yes:* _____*useful* ____*not useful*
Focused on an E-B innovation that was not complex; was easy to try out; had a perceived advantage for the users, was compatible with user culture; and its effects were observable.	_____ *Yes:* _____*useful* ____*not useful*
Use of E-B change strategies, e.g.<br>Audit/feedback	_____ *Yes:* _____*useful* ____*not useful*
An opinion leader or clinical champions	_____ *Yes:* _____*useful* ____*not useful*
QI team	_____ *Yes:* _____*useful* ____*not useful*
Clinical written/electronic reminder	_____ *Yes:* _____*useful* ____*not useful*
A written project plan	_____ *Yes:* _____*useful* ____*not useful*
An incentive (either reward or sanction)	_____ *Yes:* _____*useful* ____*not useful*
Educational sessions	_____ *Yes:* _____*useful* ____*not useful*
Educational/academic outreach/mentoring/precepting	_____ *Yes:* _____*useful* ____*not useful*
Marketing/educational materials: flyers; announcements; pamphlets; posters	_____ *Yes:* _____*useful* ____*not useful*
Expert consultants	_____ *Yes:* _____*useful* ____*not useful*
Patient activation	_____ *Yes:* _____*useful* ____*not useful*
Formal change in policy, procedure, etc.	_____ *Yes:* _____*useful* ____*not useful*
Change in official products or equipment or allowable meds	_____ *Yes:* _____*useful* ____*not useful*
Another system change =	_____ *Yes:* _____*useful* ____*not useful*
Role models	_____ *Yes:* _____*useful* ____*not useful*
Evaluation of implementation (process and progress)=	_____ *Yes:* _____*useful* ____*not useful*
Use of a demonstration unit	_____ *Yes:* _____*useful* ____*not useful*

2. <u>PROJECT-RELATED CHANGE/FACILITATORS/HINDRANCES:</u> <u>Intro by facilitator</u>: *You have talked about various forces that (either) helped, encouraged or enabled you or others to successfully make and sustain a targeted EBP change (and/or) restraining/hindering forces that inhibited an ability to implement EBP or sustain it once an initial change was made. Before we move on to the next topic, I have a checklist of such potential factors … some you have mentioned, others not. THERE IS NO RIGHT ANSWER but I just want to jog your memory and see if this list helps you recall any other factors. So could you just check all those which are true and their influence in your perception for this project.* (Follow-up prn)

Potential factors related to the Facilitation or Hindrance of your implementation efforts.	Please check all factors that **were PRESENT** and **HELPED** to implement and/or **sustain** the evidence-based change you were trying to make(check 1 or both):	Please check all factors that *were ABSENT* or *GOT IN THE WAY* of your ability to implement and/or sustain you targeted evidence-based change (check 1 or both):
Change strategies on the other sheet	**____ Implement ____Sustain (HELPED)**	____ *Implement* ____*Sustain (HINDERED)*
People leading the change: ____staff? _____manager? ____other leadership?	**____ Implement ____Sustain**	____ *Implement* ____*Sustain*
People supporting the change: __colleagues? ___managers? __other leadership? __patients	**____ Implement ____Sustain**	____ *Implement* ____*Sustain*
Managerial-clinician relations	**____ Implement ____Sustain**	____ *Implement* ____*Sustain*
Staff in key positions; role models; peer opinion leaders	**____ Implement ____Sustain**	____ *Implement* ____*Sustain*
Degree of experimentation/risk-taking in the culture	**____ Implement ____Sustain**	____ *Implement* ____*Sustain*
Other aspects of your culture	**____ Implement ____Sustain (HELPED)**	____ *Implement* ____*Sustain (HINDERED)*
External regulatory mandate or pressures	**____ Implement ____Sustain**	____ *Implement* ____*Sustain*
Knowledge/attitude/incentive of those targeted for change	**____ Implement ____Sustain**	____ *Implement* ____*Sustain*
Your monitoring/feedback/data system	**____ Implement ____Sustain**	____ *Implement* ____*Sustain*
Level of teamwork/collaboration on the task force/committee	**____ Implement ____Sustain**	____ *Implement* ____*Sustain*
Level of teamwork/collaboration with those outside the task force/committee	**____ Implement ____Sustain**	____ *Implement* ____*Sustain*
Coordination across departments	**____ Implement ____Sustain (HELPED)**	____ *Implement* ____*Sustain (HINDERED)*
Participation/involvement of staff	**____ Implement ____Sustain**	____ *Implement* ____*Sustain*
Time to do the work (where?)	**____ Implement ____Sustain**	____ *Implement* ____*Sustain*
Other resources	**____ Implement ____Sustain**	____ *Implement* ____*Sustain*
Availability of experts	**____ Implement ____Sustain**	____ *Implement* ____*Sustain*
Availability of the evidence	**____ Implement ____Sustain**	____ *Implement* ____*Sustain*
Strength of the available evidence	**____ Implement ____Sustain**	____ *Implement* ____*Sustain*
OTHER=	**____ Implement ____Sustain**	____ *Implement* ____*Sustain*

3. <u>EBP AS NORM/MOTIVATION</u>: <u>Intro by facilitator</u>: *You have talked about various forces that (either) helped, encouraged or enabled you to have the motivation to want to/implement EBP over the years (and/or) restraining/hindering forces or factors that inhibited your desire to/motivation to implement EBP or sustain work on EBP over the years. Before we move on to the next topic, I have a checklist of such potential factors … some you have mentioned, others not. THERE IS NO RIGHT ANSWER but I just want to jog your memory and see if this list helps you recall any other factors. So could you just check all those which are true in your perception for this project* (Follow-up prn)

Potential factors related to the MOTIVATION to implement EBP or not.	Please check/comment on all factors that **encouraged or were part of your department's motivation** or wish to implement EBP:	Please check/comment on all factors *that inhibited or got in the way of your department's motivation* or wish to implement EBP:
History of this organization		
Leadership (which level?)		
Strategic vision		
Managerial-clinical relations		
Staff in key positions		
Degree of experimentation/risk-taking in the culture		
Other cultural aspect/s =		
The monitoring/feedback system		
External regulatory mandate		
External opportunity		
External relationships/ partnerships		
Organizational capacity to change		
Politics		
Strategic planning process (e.g., with a situational or environmental assessment)		
Other =		
Other =		
Other =		
Other =		

4. EBP AS NORM/HOW (with typical intro by facilitator per strategies used to try to enable the routine use of evidence and discussion of their timing)

Potential STRATEGIES related to the PROCESS OF CREATING EBP AS THE NORM *(For those with multiple components, if only some apply, circle the item/s which do apply)*	*Please check "Yes" for all that apply to your effort to make EBP the routine in your department and then indicate whether you think they were useful or not*
Strategic planning:<br>1. *Environmental/situational assessment*<br>2. *Visioning*<br>3. *Identification of new, clearly articulated and supported values/beliefs*<br>4. *Identification of a new sense of purpose*<br>5. *Identification of simple, clear and shared goals*	1. ______ *Yes:* _____*useful* ____*not useful*<br>2. ______ ______ _____<br>3. ______ ______ _____<br>4. ______ ______ _____<br>5. ______ ______ _____
Use of new language	_____ *Yes:* _____*useful* ____*not useful*
Revision of priorities	_____ *Yes:* _____*useful* ____*not useful*
Creation of new capabilities	_____ *Yes:* _____*useful* ____*not useful*
Use of a research utilization/EBP model or conceptual framework	_____ *Yes:* _____*useful* ____*not useful*
Leadership expectations for change	_____ *Yes:* _____*useful* ____*not useful*
Special communication methods/media for marketing, broad education, or kick-off conferences	_____ *Yes:* _____*useful* ____*not useful*
Use of champions and opinion leaders	_____ *Yes:* _____*useful* ____*not useful*
Mandated changes	_____ *Yes:* _____*useful* ____*not useful*
External networking	_____ *Yes:* _____*useful* ____*not useful*
• *Engagement of staff for broad based EBP action*<br>• *Engagement of resistant individuals*	_____ *Yes:* _____*useful* ____*not useful*<br>_____ _____ ____
Leverage of external expectations	_____ *Yes:* _____*useful* ____*not useful*
Role modeling	_____ *Yes:* _____*useful* ____*not useful*
Use of consultant/s	_____ *Yes:* _____*useful* ____*not useful*
Demonstration projects or generating short –term wins	_____ *Yes:* _____*useful* ____*not useful*
Protection of the EBP goal despite other pressures	_____ *Yes:* _____*useful* ____*not useful*
Provision of resources/budgetary needs	_____ *Yes:* _____*useful* ____*not useful*
Consolidating gains	_____ *Yes:* _____*useful* ____*not useful*
Celebrating wins and progress	_____ *Yes:* _____*useful* ____*not useful*
Other =	_____ *Yes:* _____*useful* ____*not useful*

5. <u>EBP AS NORM/WHAT:</u> (with typical intro by facilitator relative to key contextual elements or other entities in the system changed to enhance or support the <u>routine</u> use of evidence…and discussion of their timing)

Potential ORGANIZATIONAL CHANGES in systems/routine processes/ structures/infra-structures that were implemented or created to enhance or support the <u>routine</u> use of evidence<br><br>[NOTE: *If one of these factors ALREADY EXISTED IN THE DEPARTMENT PRIOR TO THE EBP NORM INITIATIVE, please check the box]*	*Please check "Yes" for all that apply to your effort to make EBP the routine in your department and then indicate whether you think they were useful or not*
Alignment of departmental infrastructures with the goal, purpose, values, vision, strategy, and/or priorities of EBP [ ]	_____ *Yes:* _____*useful* ____*not useful*
Creation of a new process or standard approach to organizational change [ ]	_____ *Yes:* _____*useful* ____*not useful*
Changes in coordinative/collaborative mechanisms across departments or disciplines [ ]	_____ *Yes:* _____*useful* ____*not useful*
Change in various operations, i.e., in:<br>1. *Structures [ ]*<br>2. *Systems [ ]*<br>3. *Roles [ ]*<br>4. *Job descriptions [ ]*<br>5. *Performance evaluation systems [ ]*<br>6. *Processes [ ]*<br>7. *Documentation [ ]*	 1. _____ *Yes:* _____*useful* ____*not useful*<br>2. _____ _____ ____<br>3. _____ _____ ____<br>4. _____ _____ ____<br>5. _____ _____ ____<br>6. _____ _____ ____<br>7. _____ _____ ____
**Budgeting priorities *[ ]***	_____ *Yes:* _____*useful* ____*not useful*
Change in decision making structures/committee or related expectations [ ]	_____ *Yes:* _____*useful* ____*not useful*
Formalization of new practices into policies, procedures and routine systems [ ]	_____ *Yes:* _____*useful* ____*not useful*
Change in internal communication and dissemination systems [ ]	_____ *Yes:* _____*useful* ____*not useful*
New external communication linkages [ ]	_____ *Yes:* _____*useful* ____*not useful*
Integration of new values/expectations into the incentive system [ ]	_____ *Yes:* _____*useful* ____*not useful*
Creation of monitoring/feedback systems & related information technologies [ ]	_____ *Yes:* _____*useful* ____*not useful*
Focus of human resource training [ ]	_____ *Yes:* _____*useful* ____*not useful*
Other =	_____ *Yes:* _____*useful* ____*not useful*
